# Validity and reliability of quality of recovery-35 Thai version: a prospective questionnaire-based study

**DOI:** 10.1186/s12871-016-0229-7

**Published:** 2016-08-18

**Authors:** Siriporn Pitimana-aree, Suthipol Udompanthurak, Saowaphak Lapmahapaisan, Matula Tareerath, Aungsumat Wangdee

**Affiliations:** 1 Department of Anesthesiology, Faculty of Medicine Siriraj Hospital, Mahidol University, Bangkok, 10700 Thailand; 2 Clinical Epidemiology Unit, Faculty of Medicine Siriraj Hospital, Mahidol University, Bangkok, 10700 Thailand

**Keywords:** Anesthesia, Quality of recovery, Reliability, Surgery, Validity

## Abstract

**Background:**

The quality of patients’ recovery following surgery and anesthesia has been a matter of focus and concern over the past decade. The Quality of Recovery-40 (QoR-40) questionnaire was developed and validated for post-anesthesia patient evaluation. The QoR-40, however, is English-based and was tested and validated in Caucasian patients, a population that is culturally and behaviorally different from the Thai population. The objective of this study was to translate and modify the original English-language QoR-40 into the Thai language and evaluate the Quality of Recovery-35 Thai version for measuring health outcomes in Thai patients after surgery and anesthesia.

**Methods:**

Translation was performed according to internationally recognized translation standards to ensure the integrity of the translated version. Consistent with the recommendations of 25 anesthesiologists, five questions from the original QoR-40 were excluded. The 35-item questionnaire was then translated into the Thai language and renamed the Quality of Recovery-35 Thai version (Thai QoR-35). Overall, 43 outpatients and 53 inpatients rated their health and recovery status using three patient evaluation tools: 100-mm Visual Analogue Scale–Recovery (VAS-R), six-item Activities of Daily Living (ADL) questionnaire, and Thai QoR-35.

**Results:**

Overall, 90 % of patients took <10 min to complete the Thai QoR-35 questionnaire. The Thai QoR-35 and VAS-R showed good convergent validity (*r* = 0.84, *P* < 0.001). Discrimination validity was supported by a significant difference in mean scores for recovery among the Thai QoR-35, VAS-R, and ADL when compared between outpatients and inpatients (*P* < 0.01) and also between baseline and postoperative values (*P* < 0.001). The Thai QoR-35 also demonstrated good reliability with high internal consistency at three time points (Cronbach’s alpha = 0.88, 0.89, 0.91, respectively; *P* < 0.01) and a split-half reliability coefficient of 0.65 (*P* < 0.001).

**Conclusion:**

Thai QoR-35 is a valid, reliable tool for evaluating quality of recovery in Thai patients.

**Electronic supplementary material:**

The online version of this article (doi:10.1186/s12871-016-0229-7) contains supplementary material, which is available to authorized users.

## Background

Health-related quality of life has been accepted as an important clinical measure and an endpoint for comparisons in research and long-term follow-up studies. In anesthesia, the time to eye opening, time to respond to verbal commands, extubation time, and anesthesia-related adverse events are typically evaluated and compared as a measure of quality of care in many studies [1–3]. Quality of Recovery-40 (QoR-40) is a 40-item questionnaire that covers five domains of recovery outcomes, including physical comfort, emotional state, physical independence, psychological support, and pain [4]. The QoR-40 has been tested for validity, reliability, responsiveness, and clinical utility in measuring health status after surgery and anesthesia in a variety of patients and in a systematic review [4–8]. QoR-40, however, is an English-language measurement tool that has mostly been tested in Western patients. Patients from Western cultures are very different from Thai and Asian patients with regard to culture, attitude, perception, and lifestyle.

The objective of this study was to translate and modify the original English-language QoR-40 into the Thai language and evaluate the Quality of Recovery-35 Thai version (Thai QoR-35) for measuring health outcomes in Thai patients after surgery and anesthesia.

## Methods

The protocol for this prospective, questionnaire-based study was approved by the Siriraj Institutional Review Board, Faculty of Medicine Siriraj Hospital, Mahidol University, Bangkok, Thailand. The study sequence had three phases.

- *Phase I*: After receiving permission from the originator of the QoR-40 to translate and modify it, the resulting product was critically reviewed for appropriateness and suitability in Thai patients by 25 experienced anesthesiologists, each of whom has practiced anesthesia for 5–20 years. Each of the 40 questions from the QoR-40 was evaluated by each of the reviewing anesthesiologists using a three-option scoring system (1, 0, −1), with 1 indicating absolute agreement and −1 indicating absolute disagreement. Any of the 40 questions that had a mean score of < 0.5 was considered inappropriate and removed. A total of five questions were removed from the original QoR-40, as follows: Q4: able to write; Q12: feeling in control; Q33: sore mouth; Q37: feeling angry; Q39: feeling alone. Finally, 35 questions were retained for translation and for psychometric testing during development of the Thai QoR-35.
- *Phase II*: To maintain the integrity of the original QoR-40, the remaining 35 questions were translated into Thai language by an anesthesiologist. The translated version (Thai QoR-35) was then back-translated by an English translator who is fluent in Thai language and who was blinded to the original English version. The original QoR-40 and the back-translated version of the Thai QoR-35 were then compared, and no differences in meaning were observed. Accordingly, the Thai-translated 35-item questionnaire was accepted as the Thai QoR-35. Similar to the QoR-40, the Thai QoR-35 consists of five clinical outcome domains (Additional file 1). Again similar to the QoR-40, the Thai QoR-35 is divided into two parts, with five-point Likert scales used to rate patient response (positive items: 1 = none of the time, 5 = all of the time; negative items: 1 = all of the time, 5 = none of the time). Maximum and minimum scores were 175 and 35, respectively, with higher scores reflecting better quality of recovery.
- *Phase III*: After obtaining approval from our institutional review board, 96 patients who provided written informed consent were enrolled in the study. There were 43 outpatients and 53 inpatients who met our inclusion criteria: aged >18 years, American Society of Anesthesiologists (ASA) physical status I–III, and undergoing general anesthesia for elective surgery. Patients were excluded if they were unable to read or unwilling to participate in the study. Preoperative baseline data were collected. Patients were asked to describe their recovery status using the following three evaluation tools: Thai QoR-35, six-item activities of daily living (ADL) questionnaire, 100-mm visual analogue scale of recovery status (VAS-R). Patients were asked to complete each of these tools at three time points: before surgery, before discharge from the recovery room (RR), and 24 h postoperatively (PO). The ADL score [9] is a basic, simple tool for self-evaluation of independence. ADL is composed of questions that centers on six basic daily activities: bathing, dressing, toileting, transferring, continence, feeding (Appendix). The maximum and minimum ADL scores are 6 and 12, respectively, with a higher score indicating a higher level of independence. The 100-mm VAS-R is a global assessment of recovery, where one end of the scale is 0 and the other end is 100, representing the worst and the best quality of recovery, respectively. Outpatients were instructed to complete the last of the assessments at home (24 h postoperatively) and return them in the provided self-addressed envelope.

### Validity testing

We used the following methods to evaluate validity: (1) discrimination validity, for which we compared the mean change in the Thai QoR-35 in outpatients and inpatients at two recovery time points; (2) convergent validity, for which we analyzed correlations between the Thai QoR-35 and VAS-R and the Thai QoR-35 and ADL.

### Reliability testing

Reliability was assessed by the internal consistency (Cronbach’s alpha) of the Thai QoR-35 at the three time points. Correlation between the two segments of the Thai QoR-35 (split-half correlation) was also analyzed.

### Statistical analysis

We hypothesized that the recovery score of outpatients would be better than that of inpatients. Using power analysis, a sample size of 40 patients per group was calculated to detect a type I error of 0.05 and a type II error of 0.2. This sample size could also demonstrate a mean difference in a Thai QoR35 score of 10 between outpatients and inpatients. Data were reported as means ± standard deviation (SD) or the median, range, and confidence interval. Associations were measured using Pearson’s correlation coefficient (r), Spearman’s correlation coefficient (rho), or Cronbach’s alpha (α). Changes in the Thai QoR-35 score among the three survey time points were compared using a t-test or Mann–Whitney U-test. Data were analyzed using SPSS Statistics, version 11.5 (SPSS, Chicago, IL, USA).

## Results

A total of 96 patients (53 inpatients, 43 outpatients) participated in this study. Patients’ and clinical characteristics are shown in Table 1. There were no relevant differences in sex, mean age, or ASA status between the two groups. Time to eye opening and time to orientate were not significantly different. Types and durations of surgery were different between the groups. Inpatients underwent more invasive and/or more complex procedures than outpatients. Intra-abdominal surgery (both upper and lower abdomen) was more common among inpatients, whereas less invasive procedures (e.g., hernioplasty, hemorrhoidectomy) were performed more commonly among outpatients. Breast surgery (lumpectomy or excision of a breast mass) was also common in outpatients. Duration of the operation was almost twice as long for inpatient procedures as for outpatient procedures, reflecting the differences in the nature of the surgical procedures between the groups. Although Thai QoR-35 has 35 questions, our study subjects took <10 min to complete it. The mean times were 4.8 (SD 2.3), 4.6 (SD 2.1), and 4.4 (SD 3.0) min at baseline, RR, and 24 h PO, respectively.

**Table 1:** Patients and clinical characteristics

Characteristic	IP (*n* = 53)	OP (*n* = 43)	*P*
Age (years)	37.8 (11.6)	40.8 (9.9)	0.82
Sex (F/M) (% female)	41/12 (85)	39/4 (91)	0.81
ASA status I/II	45/8	38/5	0.62
Type of surgery			
General	30	22	
Head/neck/breast	3	19	
Ear/nose/throat	7	1	
Orthopedic	13	1	
Operative time (min)	98.7 (61.6)	46.0 (39.3)	<0.001
Time to open eyes (min)	7.6 (5.1)	6.8 (5.6)	0.51
Time to orientate (min))	10.8 (6.0)	9.5 (5.5)	0.28

The results are given as the mean (SD) or the number of patients, unless otherwise stated

*IP* inpatients, *OP* outpatients

**Fig. 1:**
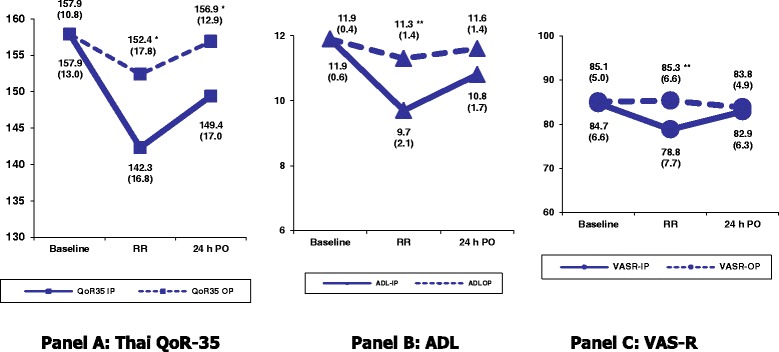
Changes in the mean score (SD) of *QoR-35*, *ADL*, and *VASR* over three time points. *p < 0.05 and **p < 0.001 compared between inpatients (IP) and outpatients (OP). *QoR-35,* Thai Quality of Recovery 35; *ADL,* activities of daily living; *VASR*, visual analogue scale of recovery; *Baseline* before surgery; *RR* before discharge from recovery room; *24 h PO* at 24 h postoperatively

The three recovery scores (ThaiQoR-35, VAS-R, ADL) for inpatients and outpatients are shown in Table 2. Baseline values of the three recovery scores were not different between the two groups. Thai QoR-35 scores at both postoperative times were significantly lower for the inpatients than the outpatients. Also, Thai QoR-35 scores at both postoperative times (142.9, SD 16.8; 149.4, SD 17.0) for the inpatients were significantly different compared to the baseline value (157.9, SD 13.0) (all *P* < 0.001). In contrast, there was no significant difference in the Thai QoR-35 scores at 24 h PO (156.9, SD 12.9) in the outpatients compared to baseline (157.9, SD 10.8). These results reflect the discrimination validity of the Thai QoR-35 score, which was able to differentiate the major and minor changes in these scores in inpatients and outpatients. Nevertheless, VAS-R and ADL scores showed a significant difference only at the RR time point. Changes in these three recovery scores over time (three time points) are shown in Fig. 1. The Thai QoR-35 score at RR was markedly decreased in the inpatients. Their recovery had not resumed baseline status at 24 h PO, whereas it had in the outpatients.

**Table 2:** Changes in three recovery scores (Thai QoR-35, VAS-R, ADL) at three time points: inpatients vs. outpatients

Questionnaire	IP^a^	OP^a^	Change^b^	*P*
Thai QoR-35 (175^c^)				
Baseline	157.9 (13.0)	157.9 (10.8)	−0.001 (−4.9 to 4.9)	1
RR	142.9 (16.8)	152.4 (17.8)	−10.1 (−17.1 to −3.1)	0.005
24 h PO	149.4 (17.0)	156.9 (12.9)	−7.5 (−13.8 to −1.0)	0.015
VAS-R (100^c^)				
Baseline	84.7 (6.6)	85.1 (5.0)	−0.4 (−2.8 to 2.0)	0.718
RR	78.8 (7.7)	85.3 (6.6)	−6.5 (−9.5 to −3.6)	<0.001
24 h PO	82.9 (6.3)	83.8 (4.9)	−0.9 (−3.2 to 1.4)	0.432
ADL (12^c^)				
Baseline	11.9 (0.4)	11.9 (0.6)	0.03 (−0.2 to 0.2)	0.802
RR	9.7 (2.1)	11.3 (1.4)	−1.6 (−2.4 to 0.9)	<0.001
24 h PO	10.8 (1.7)	11.6 (1.4)	−0.75 (−1.4 to 0.1)	0.19

*Thai QoR-35* Thai quality of recovery 35; *VAS-R* visual analogue scale of recovery; *ADL* activities of daily living; *Baseline* before surgery and anesthesia; *RR* before discharge from recovery room; *24 h PO* 24 h postoperatively

^a^Mean (SD)

^b^Mean (95 % confidence interval)

^c^Total score

Thai QoR-35 consists of five domains: physical comfort, emotional state, psychological support, physical independence, and pain (Table 3). At baseline, emotional state showed the worst score and physical independence the best. Conversely, physical independence followed by physical comfort scores had major decreases from baseline to both the RR time point and 24 h PO. All scores of every domain were better for the outpatients than for the inpatients.

**Table 3:** Domain scores (%)

Parameter	Physical comfort	Emotional state	Psychological support	Physical independence	Pain
	(60)^a^	(35)^a^	(30)^a^	(20)^a^	(30)^a^
At baseline					
IP	54.0 (90.0)	30.1 (86.0)	28.0 (93.3)	19.2 (96.0)	26.5 (88.3)
OP	54.0 (90.0)	30.0 (85.7)	28.2 (94.0)	19.7 (98.5)	26.4 (88.0)
At RR					
IP	50.6 (84.3)	29.0 (82.9)	25.0 (83.3)	13.3 (66.5)	24.2 (80.6)
OP	53.0 (88.3)	31.0 (88.6)	27.5 (91.6)	16.9 (84.5)	24.0 (80.0)
At 24 h PO					
IP	51.7 (85.6)	29.7 (84.9)	27.4 (91.3)	16.1 (80.5)	24.3 (81.0)
OP	54.6 (91.0)	31.0 (88.5)	28.0 (93.3)	18.4 (92.0)	25.0 (83.3)

Results are shown as the score (%)

*IP* inpatients, *OP* outpatients or ambulatory patients; *baseline* before surgery and anesthesia; *RR* before discharge from recovery room; *24 h PO* 24 h postoperatively

^a^Maximum score

The correlation between Thai QoR-35 and VAS-R was significant (Table 4), and it was consistent throughout the three time points (*r* = 0.77, *r* = 0.82, *r* = 0.84; all *P* < 0.001). As ADL mainly measures physical or functional ability, the correlation between Thai QoR-35 and ADL showed only low to moderate correlation over the three time points (*r* = 0.21, *r* = 0.50, *r* = 0.29 and *P* = 0.04, *P* < 0.001, *P* = 0.004, respectively). ADL and the physical independence domain of Thai QoR-35, however, showed a strong correlation (*r* = 0.5–0.7; *P* < 0.001).

**Table 4:** Correlation coefficient of Thai QoR-35 with VAS and ADL

Thai QoR-35	Correlation coefficient r (*P*)	
	VAS-R	ADL
Baseline	0.77 (<0.001)	0.21 (0.04)
RR	0.82 (<0.001)	0.50 (<0.001)
24 h PO	0.84 (<0.001)	0.29 (0.004)

*Thai QoR-35* Thai quality of recovery 35; *VAS-R* visual analogue scale of recovery; *ADL* activities of daily living; Baseline before surgery and anesthesia; *RR* before discharge from recovery room; *24 h PO* 24 h postoperatively

Our study did not perform test–retest reliability due to an expectation of recall bias and differences in health status during the three time points (baseline, RR, 24 h PO). Internal consistency of Thai QoR-35 at the three time points were high: Cronbach’s alpha = 0.88, 0.89, 0.91 (*P* < 0.01), respectively. The correlation between the two segments of the Thai QoR-35 (split-half correlation) was moderate (*r* = 0.65; *P* < 0.001). These data confirm the reliability of the Thai QoR-35.

## Discussion

The QoR-40 was translated into the Thai language and culturally adapted to a Thai population. The resulting Thai QoR-35 was then tested for validity (a measure of accuracy) and reliability (a measure of consistency). Our primary hypothesis for testing validity was that Thai QoR-35 could discriminate recovery status between inpatients and outpatients. The results showed a significant difference in Thai QoR-35 scores between inpatients and outpatients and between baseline and two postoperative time points. Convergent validity showed a high correlation between Thai QoR-35 and VAS-R. The high correlation coefficient of Thai QoR-35 indicated good reliability. All 96 patients fully participated in all aspects of the study, with most subjects completing the Thai QoR-35 within 5 min. These parameters indicated the good acceptability and practicability of QoR-35.

The ability to measure quality of care has direct benefit to patients and facilitates auditing and improving health care-related services and protocols. Although the Aldrete scoring system has been widely adopted as an anesthesia recovery score, it addresses only the physiologic dimension and does not evaluate overall recovery outcomes. A credible instrument for measuring quality of recovery should be studied and accepted as valid and reliable, however, and the Aldrete scoring system has never been validated. At present, there is no generally accepted gold standard for measuring quality of recovery.

Other quality of recovery scores, including the Quality of Recovery 9 (QoR-9) and QoR-40 [4, 10], the 24-h Functional Ability Questionnaire (24hFAQ) [11], and the Postoperative Quality Recovery Scale (PQRS) [12] have been recently studied and validated. The 24hFAQ was developed to measure final recovery and satisfaction 24 h after surgery. It consists of 21 questions and mainly focuses on cognitive, physical, and satisfaction domains. The 24hFAQ, however, was validated only in an outpatient setting, so its application in an inpatient surgical population might be questioned.

Royse and colleagues [12] attempted to develop the Postoperative Quality Recovery Scale (PQRS), which also incorporates physiologic assessment. Although the PQRS was administered in a wide range of populations and in several languages, the investigators found that a considerable number of patients (including young children) within the studied populations could not or refused to complete the test, particularly during the early postoperative period. In contrast, none of the patients in the present study refused to participate, with nearly all patients completing the questionnaire within 5 min.

Chan and colleagues [13] conducted a psychometric test on the Chinese version of the QoR-9 and found nearly perfect agreement between the Chinese and English versions. The QoR-9 is a simple instrument that contains only nine questions, but it lacks detail. The QoR-40 elicits more patient information with minimal training, need for assistance, and time needed to complete the questionnaire. In addition to the QoR-40 being widely tested, it is a highly significant predictor of quality of life at late recovery. It is also appropriate for use in research and for quality assurance testing [4–8]. It is for these reasons that we chose to translate the QoR-40 in our study.

Unlike the study by Chan et al. [13], we modified the original QoR-40 by deleting five questions based on the professional advice of 25 experienced anesthesiologists. The following five questions were excluded from the translated version: Q4: able to write; Q12: feeling in control; Q33: sore mouth; Q37: feeling angry; Q39: feeling alone. These questions were excluded to improve the cultural suitability of the test, thereby adapting the test to the lifestyle of the Thai people. The Thai family structure is an extended family, with patients being attended to by family members who stay with them and/or take care of them at home and/or at the hospital. Regarding a sense of control, many Thai patients do not desire or seek control in a health crisis setting. Rather, they prefer to give up control and have someone take responsibility for their care when they are ill. In addition, Thai people rarely outwardly express anger, they normally avoid conflict, and they prefer listening to reading and writing. Among the Thai patient population and based on our postoperative data, sore mouth is not a common problem. Each of the deleted items is not appropriate for the Thai culture and lifestyle.

Our study demonstrated the feasibility, practicability, applicability, validity, and reliability of the Thai QoR-35. This QoR-35 may be generalizable to other Asian populations that have close or similar cultures and lifestyles. This tool and its potential transferability to other countries could expand the opportunity for multi-country collaborative research and quality audits, which may be the strength of our study. There are, however, some limitations to it as well. First, we tested the Thai QoR-35 in a small number of patients and followed those patients for only 24 h PO. Additional studies with larger study populations that follow patients for longer than 24 h after surgery should be undertaken. Second, further studies are needed to evaluate the utility of the QoR-35 across a wide range of ages, surgical types, and Asian populations.

## Conclusions

This study confirmed the validity and reliability of the Thai QoR-35 as a tool for assessing the quality of recovery in Thai surgical patients. Other ethnic populations for whom postoperative recovery scores have to be adapted to cultural and lifestyle characteristics may benefit from the methods and data reported in this study.

## Appendix

**Table 5:** ADL: index of indepence in activity of daily living

Activity point (2 or 1)	Independence (2 points)	Dependence (1 point)
	NO supervised, direction or personal assistance	WITH supervised, direction or personal assistance
Bathing	Bathes self completely in bathing or need help only single part of body.	Needs help with bathing more than one part of the body, geting out of the shower. Requires total bathing
Dressing	Self gets clothes from closet or drawer and puts on clothes completely.	Needs help with dressing self or needs to be completely dressed.
Toileting	Goes to toilet, gets on and off, arranges clothes, cleans genital area without help.	Needs help transferring to the toilet, cleaning self or uses bedpan or commode.
Transferring	Moves in and out of bed or chair unassisted.	Needs help in moving from bed to chair or requires a complete transfer.
	Mechanical transfer aids are acceptable.	
Continence	Exercises complete self control over urination and defecation.	Is partially or totally incontinent of bowel or bladder.
Feeding	Gets food from plate into mouth without help.	Needs partial or total help with feeding.

(Maximum 12 points, Minimum 6 points)

Total Points

Reference: Adapts from Katz index of independence in activity daily living (Urol Nurs 2007;27: 93-4)

## Additional file

Additional file 1: Thai QoR 35 questionnaire. (DOCX 40 kb)

## Abbreviations

24 h PO: 24h postoperatively
24hFAQ: 24-h functional ability questionnaire
ADL: Activities of daily living
ASA: American Society of Anesthesiologists
PQRS: Postoperative quality recovery scale
QoR-40: Quality of recovery 40
r: Pearson’s correlation coefficient
RR: Recovery room (at the time of discharge)
VAS-R: Visual analogue scale of recovery.
