# Supplementary material for: Validity and reliability of quality of recovery-35 Thai version: a prospective questionnaire-based study
Source: BMC Anesthesiol. 2016 Aug 18;16:64. doi: 10.1186/s12871-016-0229-7 (PMC4990847; doi:10.1186/s12871-016-0229-7)
Supplement: Additional file 1: — Thai QoR 35 questionnaire. (DOCX 40 kb) [file 12871_2016_229_MOESM1_ESM.docx]

**Appendix 1(QoR35)**

**แบบสอบถามคุณภาพการฟื้นจากการระงับความรู้สึก (The Thai QoR-35)**

แบบสอบถามนี้จัดทำขึ้นเพื่อใช้ประเมินคุณภาพการฟื้นจากการระงับความรู้สึกของท่าน

คำถามทั้งหมดมี 35 ข้อ แบ่งเป็น 2 ส่วนใหญ่ๆ คือ ส่วน ก. และส่วน ข.

ส่วน ก. จำแนกออกเป็น 4 หมวด และประกอบด้วยคำถาม 16 ข้อ

ส่วน ข. จำแนกออกเป็น 3 หมวด และประกอบด้วยคำถาม 19 ข้อ

ขอให้ท่านตอบแบบสอบถามตรงกับความรู้สึกที่เกิดขึ้นจริงในขณะนั้น และ

ถ้าเป็นไปได้ ขอให้ท่านตอบคำถามทุกข้อ เพื่อเป็นประโยชน์แก่การศึกษา

และการพัฒนาคุณภาพในการดูแลรักษาผู้ป่วยต่อไป

**ตัวอย่าง** ถ้าท่านหายใจได้สะดวกตลอดเวลา ท่านจะเลือกตัวเลือกที่ **5** ดังแสดงด้านล่าง

ไม่เคย เกิดขึ้นบ้าง เกิดขึ้นบ่อยๆ เกิดขึ้นเกือบ เกิดขึ้น

เกิดขึ้นเลย ตลอดเวลา ตลอดเวลา

(None of (Some of (Usually) (Most of (All of

the time) the time) the time) the time)

ท่านรู้สึกหายใจได้สะดวก 1 2 3 4 5

(Able to breathe easily)

**ส่วน ก. มี 16 คำถาม และในแต่ละข้อคำถามจะมีตัวเลือก 5 ตัวเลือก**

**(Part A consists of 16 questions, each has 5 choices)**

**(คะแนน 1ถึง 5: 1 = ไม่เคยเกิดขึ้นเลย และ 5 = เกิดขึ้นตลอดเวลา)**

**(score1-5; vary from 1= None of the time to 5= All of the time)**

**ขอให้ตอบคำถามตรงกับความรู้สึกที่เกิดขึ้นจริง โดยการทำเครื่องหมายกากบาท ( X ) บนหมายเลขที่ท่านเลือก**

ไม่เคย เกิดขึ้นบ้าง เกิดขึ้นบ่อยๆ เกิดขึ้นเกือบ เกิดขึ้น

เกิดขึ้นเลย ตลอดเวลา ตลอดเวลา

(None of (Some of (Usually) (Most of (All of

the time) the time) the time) the time)

**หมวด 1 ความรู้สึกสบายกาย**

**(Dimension1A Comfort)**

1.1 ท่านหายใจได้สะดวก 1 2 3 4 5

(Able to breathe easily)

ไม่เคย เกิดขึ้นบ้าง เกิดขึ้นบ่อยๆ เกิดขึ้นเกือบ เกิดขึ้น

เกิดขึ้นเลย ตลอดเวลา ตลอดเวลา

(None of (Some of (Usually) (Most of (All of

the time) the time) the time) the time

1.2 ท่านนอนหลับได้ดี 1 2 3 4 5

(Have a good sleep)

1.3 ท่านรู้สึกเจริญอาหาร 1 2 3 4 5

(Been able to enjoy food)

1.4 ท่านรู้สึกสงบนิ่ง 1 2 3 4 5

(Feel calm)

**หมวด 2 ความรู้สึกทางอารมณ์**

**(Dimension2A Emotions)**

2.1 ท่านรู้สึกเป็นปกติ 1 2 3 4 5

(Having a feeling of

general well-being)

2.2 ท่านรู้สึกสบาย 1 2 3 4 5

(Feeling comfortable)

**หมวด 3 ความรู้สึกทางกายภาพ**

**(Dimension3A Physical Independence)**

3.1 ท่านพูดได้เป็นปกติ 1 2 3 4 5

(Have normal speech)

3.2 ท่านดูแลทำความสะอาดตัวเองได้ 1 2 3 4 5

(Able to wash, brush teeth

or shave)

3.3 ท่านดูแลการแต่งตัวของตัวเองได้ 1 2 3 4 5

(Able to look after your

own appearance)

3.4 ท่านทำกิจวัตรประจำวันได้ 1 2 3 4 5

(Able to return to work

or usual home activities)

ไม่เคย เกิดขึ้นบ้าง เกิดขึ้นบ่อยๆ เกิดขึ้นเกือบ เกิดขึ้น

เกิดขึ้นเลย ตลอดเวลา ตลอดเวลา

(None of (Some of (Usually) (Most of (All of

the time) the time) the time) the time)

**หมวด 4 ความช่วยเหลือ**

**(Dimension4A Patient Support)**

4.1 ท่านสามารถติดต่อกับเจ้าหน้าที่

โรงพยาบาลได้

(ในขณะอยู่โรงพยาบาล) 1 2 3 4 5

(Able to communicate with

hospital staff; when in hospital)

4.2 ท่านสามารถติดต่อกับเพื่อน

หรือญาติได้ 1 2 3 4 5

(Able to communicate with

family and friends)

4.3 ท่านได้รับความดูแลช่วยเหลือจาก

แพทย์(ในขณะอยู่โรงพยาบาล) 1 2 3 4 5

(Getting support from hospital

doctors; when in hospital)

4.4 ท่านได้รับความดูแลช่วยเหลือจาก

พยาบาล(ในขณะอยู่โรงพยาบาล) 1 2 3 4 5

(Getting support from hospital

nurses; when in hospital)

4.5 ท่านได้รับความดูแลช่วยเหลือ

จากเพื่อน หรือญาติ 1 2 3 4 5

(Having support from

family or friends)

4.6 ท่านเข้าใจคำแนะนำ และ /

หรือ คำสั่งทางแพทย์ 1 2 3 4 5

(Able to understand

instructions or advice)

**ส่วน ข. มี 19 คำถาม และในแต่ละข้อคำถามจะมีตัวเลือก 5 ตัวเลือก**

**(Part B consists of 19 questions, each has 5 choices)**

**(คะแนน 1ถึง5: 1 = ไม่เคยเกิดขึ้นเลย และ 5 = เกิดขึ้นตลอดเวลา)**

**(score1-5; vary from 1= None of the time to 5= All of the time)**

ขอให้ตอบคำถามที่ตรงกับความรู้สึกที่เกิดขึ้นจริงโดยการทำเครื่องหมายกากบาท (X) บนหมายเลขที่ท่านเลือก

**ตัวอย่าง** ถ้าท่านไม่มีอาการคลื่นไส้เลยตลอดระยะเวลา ท่านจะเลือกตัวเลือกเลข **5** ดังแสดงด้านล่าง

ไม่เคย เกิดขึ้นบ้าง เกิดขึ้นบ่อยๆ เกิดขึ้นเกือบ เกิดขึ้น

เกิดขึ้นเลย ตลอดเวลา ตลอดเวลา

(None of (Some of (Usually) (Most of (All of

the time) the time) the time) the time)

อาการคลื่นไส้ 5 4 3 2 1

(Nausea)

ไม่เคย เกิดขึ้นบ้าง เกิดขึ้นบ่อยๆ เกิดขึ้นเกือบ เกิดขึ้น

เกิดขึ้นเลย ตลอดเวลา ตลอดเวลา

(None of (Some of (Usually) (Most of (All of

the time) the time) the time) the time)

**หมวด 1 ความรู้สึกสบายกาย**

**(Dimension1B Comfort)**

1.1 อาการคลื่นไส้ 5 4 3 2 1

(Nausea)

1.2 อาการอาเจียน 5 4 3 2 1

(Vomiting)

1.3 อาการขย้อน 5 4 3 2 1

(Dry retching)

1.4 อาการกระสับกระส่าย 5 4 3 2 1

(Feeling restless)

1.5 อาการสั่นกระตุก 5 4 3 2 1

(Shaking or twitching)

1.6 อาการสั่นทั่วตัว 5 4 3 2 1

(Shivering)

1.7 อาการหนาวสั่น 5 4 3 2 1

(Feeling too cold)

1.8 อาการวิงเวียน 5 4 3 2 1

(Feeling dizzy)

ไม่เคย เกิดขึ้นบ้าง เกิดขึ้นบ่อยๆ เกิดขึ้นเกือบ เกิดขึ้น

เกิดขึ้นเลย ตลอดเวลา ตลอดเวลา

(None of (Some of (Usually) (Most of (All of

the time) the time) the time) the time)

**หมวด 2 ความรู้สึกทางอารมณ์**

**(Dimension2B Emotions)**

2.1 ฝันร้าย 5 4 3 2 1

(Had bad dream)

2.2 ท่านรู้สึกวิตกกังวล 5 4 3 2 1

(Feeling anxious)

2.3 ท่านรู้สึกซึมเศร้า 5 4 3 2 1

(Feeling depressed)

2.4 ท่านรู้สึกหลับยาก 5 4 3 2 1

(Had difficulty

falling asleep)

2.5 ท่านรู้สึกสับสน 5 4 3 2 1

(Feeling confused)

**หมวด 3 ความเจ็บปวด**

**(Dimension 3B Pain)**

3.1 ความเจ็บปวดระดับปานกลาง 5 4 3 2 1

(Moderate pain)

3.2 ความเจ็บปวดระดับรุนแรง 5 4 3 2 1

(Severe pain)

3.3 อาการปวดศีรษะ 5 4 3 2 1

(Headache)

3.4 อาการปวดกล้ามเนื้อ 5 4 3 2 1

(Muscle pain)

3.5 อาการปวดหลัง 5 4 3 2 1

(Backache)

3.6 อาการเจ็บคอ 5 4 3 2 1

(Sore throat)

**ท่านใช้เวลาในการตอบแบบสอบถามนาน……………………นาที**

(How much time do you take to complete the questionnaire? ……….. minutes
